# Exploring the Role of TaERF4a in Enhancing Drought Tolerance and Regulating Dehydrin *WZY1-2* Gene Expression in Wheat

**DOI:** 10.3390/plants14081214

**Published:** 2025-04-15

**Authors:** Ying Yang, Xinfei Li, Qinying Li, Wenqiang Li, Aina Wang, Hao Liu

**Affiliations:** 1 College of Nursing, Weinan Vocational & Technical College, Weinan 714026, China; yy820923@sina.com; 2 College of Agricultural and Forestry, Weinan Vocational & Technical College, Weinan 714026, China; 19894688168@163.com; 3 State Key Laboratory for Crop Stress Resistance and High-Efficiency Production, College of Life Sciences, Northwest A&F Unicersity, Yangling 712100, China; li15725480698@163.com (Q.L.); wqli@nwafu.edu.cn (W.L.); 4 College of Horticulture, Ludong University, Yantai 264000, China

**Keywords:** dehydrin, *TaERF4a*, virus-induced gene silencing, transactivation, dual-luciferase assay

## Abstract

Dehydrins (DHNs) belong to the second family of late embryogenesis abundant (LEA) proteins, which are widely distributed in plants. We cloned a SK_3_-type DHN gene named *WZY1-2* in Zheng yin 1 cultivar of *Triticum aestivum*. An ERF-type transcription factor TaERF4a was found to be involved in the regulation of the dehydrin *WZY1-2* gene in our last report. The stress-responsive ability and dual-luciferase assay demonstrated that TaERF4a positively regulates *WZY1-2* gene transcription under stress conditions. In this study, we further characterized the role of the transcription factor TaERF4a in plant drought tolerance. *Arabidopsis thaliana* heterologously overexpressing *TaERF4a* exhibited higher survival rate, increased superoxide dismutase (SOD) activity, elevated proline and chlorophyll content, and reduced malondialdehyde (MDA) content under drought conditions. Conversely, silencing *TaERF4a* in Chinese spring wheat using the virus-induced gene silencing (VIGS) method increased the sensitivity of plants to drought stress. Furthermore, we identified the specific binding site of TaERF4a in the *WZY1-2* promoter. Electrophoretic mobility shift assay (EMSA) and dual-luciferase reporter assay demonstrated that TaERF4a activates the expression of the *WZY1-2* dehydrin gene through binding to the DRE *cis*-element in its promoter. Taken together, the results of our study indicate that TaERF4a positively regulates the expression of the dehydrin *WZY1-2* gene and enhances drought tolerance in plants.

## 1. Introduction

As an important staple food crop worldwide, wheat often suffers from many kinds of abiotic stresses during its growth and development periods, which seriously affects yield and quality of wheat. Late embryogenesis abundant (LEA) proteins are a group of stress proteins produced by many organisms under adverse conditions. Dehydrins (DHNs) are members of the second subfamily of LEA proteins that can be classified into five subgroups—Y_n_SK_n_, Y_n_K_n_, SK_n_, K_n_, and K_n_S—based on the number and arrangement of Y-, S-, and K-segments [1]. Due to their richness in polar hydrophilic amino acids, DHNs have a wide range of biological functions, such as preventing cell dehydration, stabilizing cell membranes, binding metal ions, and scavenging hydroxyl radicals [2]. In plants, DHNs are typically found during the late embryonic development of plant seeds and vegetative tissues when subjected to various adversities, including drought, high salinity, low/high temperatures, and other abiotic stresses [3]. The wheat dehydrin WZY1-2 investigated in this study is a SK_3_-type dehydrin belonging to the SK_n_ type.

AP2/ERF proteins are a superfamily of transcription factors widely found in plants. They play important roles in regulating plant growth and development, as well as in responding to abiotic stresses. According to the differences in conserved domains and binding sequences, AP2/ERF transcription factors are divided into five subfamilies: AP2, ERF, DREB, RAV, and Soloist, all of which contain at least one AP2 domain [4,5]. The unique AP2/ERF domain, consisting of approximately 60–70 amino acids, can recognize and participate in DNA binding through two conserved amino acid sequences: the YRG element and the RAYD element [6]. These two elements form the typical three-dimensional (3D) structure within the AP2 domain, which consists of three β-sheets and one α-helix. They specifically bind to different *cis*-acting elements and interact with other transcription factors (TFs) to regulate gene expression. In plants, AP2/ERFs function as key regulators in multiple abiotic stress responses and hormone signaling pathways [7].

Plants are often subjected to various abiotic environmental stresses, such as drought, high salt, and low temperature during their growth and development. Studies have shown that AP2/ERF transcription factors play an important role in plant resistance to abiotic stress. In particular, the DREB and ERF subfamilies are closely related to abiotic stress. For example, *TaDREB*, *OsDREB1A*, *GhDBP3*, and *GmDREB2* genes were all induced by drought, high salt, and low temperature [8,9,10,11]. Overexpression of *OsDREB1A* and *GmDREB2* genes in *Arabidopsis* improved the resistance of transgenic lines to drought and high salt stress [9,11]. The nuclear-localized protein ERF1 acts as a transcriptional activator that regulates the expression of stress-responsive genes by binding to their GCC box or DRE/CRT elements to mediate physiological responses, including proline biosynthesis and altered stomatal aperture, to enhance tolerance to different stresses [12]. Additionally, ERFs function as either positive or negative regulators of gene transcription [13]. For instance, overexpression of *AtERF2* and *AtERF4* in *Arabidopsis thaliana* revealed that AtERF2 acts as a positive regulator, while AtERF4 functions as a negative regulator of JA-responsive defense gene expression [14].

Although DHNs exhibit a variety of cell protective functions, the molecular mechanisms governing their expression have been gradually elucidated in recent years. Some studies reported that dehydrin genes can be regulated by bHLH, ABF, and DREB transcription factors. The drought stress-related bHLH transcription factor TabHLH49 positively regulated the expression of the dehydrin *WZY2* gene and improved drought tolerance of wheat [15]. In grape, VviDREBA1-1 was involved in the transcriptional activation of the *VviDHN2* gene by binding to the CRT *cis*-acting element under high CO_2_ levels to maintain table grape quality during storage at 0 °C [16]. Similarly, MsABF2 enhanced alfalfa tolerance to aluminum stress by positively regulating the expression of the *MsDHN1* gene [17]. Another research in rice reported that transcription factor OsbZIP23 directly and positively regulated the expression and H3K4me3 modification levels of dehydrin genes under drought stress [18]. In a recent study, the WRKY transcription factor AmWRKY45 was reported to regulate the SK_n_-type dehydrin AmDHN4 from *Ammopiptanthus mongolicus*, enhancing the tolerance of *Arabidopsis* to low temperature and osmotic stress [19].

Therefore, we will examine other DHNs to gain a more comprehensive understanding of their regulatory mechanisms. In our previous experiment, we identified an AP2/ERF transcription factor, TaERF4a (GenBank NO.AFP49822.1), that interacts with the wheat dehydrin *WZY1-2* gene promoter through a yeast one-hybrid assay [20]. Its positive regulation of the *WZY1-2* gene expression was confirmed by dual-luciferase transient expression assay analysis *in planta*. In this study, we will further explore the role of the TaERF4a transcription factor in plants under abiotic stress and its mechanism underlying transcriptional regulation of the dehydrin *WZY1-2* gene in wheat.

## 2. Results

### 2.1. Overexpression of TaERF4a Enhances Drought Tolerance in Transgenic Arabidopsis thaliana

**Figure 1:**
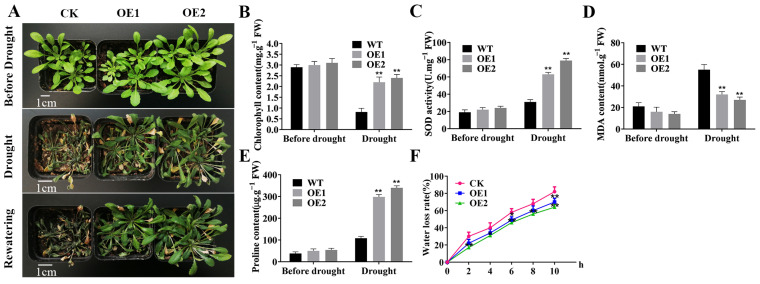
Performance of wild-type (WT) and *TaERF4a*-overexpressing *Arabidopsis* under drought and rehydration treatments; (**A**) phenotypic analysis of transgenic *Arabidopsis* plants under normal growth, drought stress conditions and after rewatering; chlorophyll content (**B**), SOD content (**C**), malondialdehyde content (**D**), and proline content (**E**) of wheat leaves from WT and transgenic plants under normal growth or drought stress conditions; and (**F**) leaf water loss rate of transgenic and WT plants after 10 h of drought treatment; data are presented as means ± SD of three replicates (* *p* < 0.05, ** *p* < 0.01).

To investigate the gene function of *TaERF4a*, *Arabidopsis* plants transformed with *TaERF4a* were used to identify its role in abiotic stress tolerance. The drought resistance of transgenic plants was evaluated at the vegetative growth stage. Before drought treatment, the growth phenotype of the *TaERF4a*-overexpressing plants at the 3-week-old seedling stage was not significantly different from that of the control plants. Next, the plants were exposed to drought stress by withholding water for 12 days. The leaves of the wild-type (WT) plants were severely wilted, whereas those of the *TaERF4a*-overexpressing plants were less affected. After the dehydration treatment, all plants were rewatered for 3 days. The WT plants still exhibited the leaf-wilting phenotype and eventually died, whereas the transgenic plants were less damaged, and most of them recovered growth (Figure 1A).

**Figure 2:**
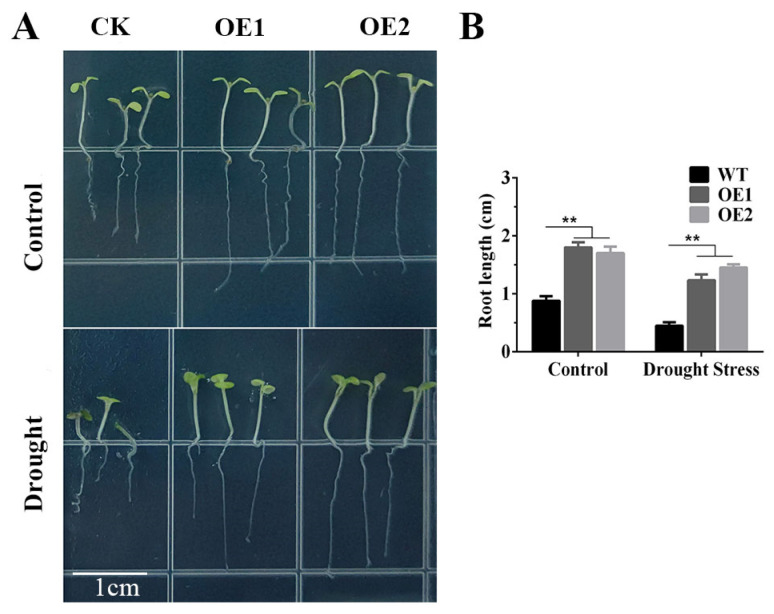
Growth status of wild-type (WT) and TaERF4a-overexpressing Arabidopsis seedlings under PEG-induced stress; (**A**) the seedlings of WT and transgenic plants were grown on 1/2 MS agar medium containing 10% PEG6000; and (**B**) the root length were measured at about 1-week-old seedling stage. Data are presented as means ± SD of three replicates (** *p* < 0.01).

We also measured the root lengths of *TaERF4a*-overexpressing plants after osmotic stress triggered by PEG treatment. After sowing the seeds of WT and transgenic lines on 1/2 MS agar medium for 5 days, significant differences in root length were observed between the WT and transgenic lines. Moreover, after 7 days of cultivation on 1/2 MS agar medium containing 10% PEG6000, the root growth and development of both WT and transgenic seedlings were inhibited under the same treatment. The WT seedlings exhibited slower growth and shorter root lengths compared to those of the transgenic plants (Figure 2), indicating that the expression of TaERF4a improved root growth of the transformed lines under stress.

### 2.2. Measurement of Physiological Indices in TaERF4a-Overexpressing Plants Under Drought Stress

We further evaluated chlorophyll content, SOD activity, proline content, and MDA content in WT and *TaERF4a*-overexpressing plants under both normal and stress conditions (Figure 1B–E). The plants subjected to drought stress exhibited considerable increases in SOD activity and proline content in both WT and *TaERF4a*-overexpressing plants, but the increase was more pronounced in transgenic plants. The MDA content was significantly lower in transgenic plants compared to WT plants. Additionally, the water loss rate was measured in detached leaves from 4-week-old WT and *TaERF4a*-overexpressing plants. After being treated for 10 h, the water loss rates in transgenic lines were less than 72.3%, while that in the WT reached up to 84.4% (Figure 1F). This indicated that the transgenic lines had a lower water loss rate and stronger drought tolerance. Additionally, the chlorophyll content was higher in the leaves of *TaERF4a*-overexpressing lines than in WT plants. Therefore, the expression of TaERF4a appears to improve the tolerance of transgenic *Arabidopsis* to drought stress.

### 2.3. Silencing of the TaERF4a Gene Affects the Phenotype and Physiological Indices of Wheat Under Drought Stress Treatment

**Figure 3:**
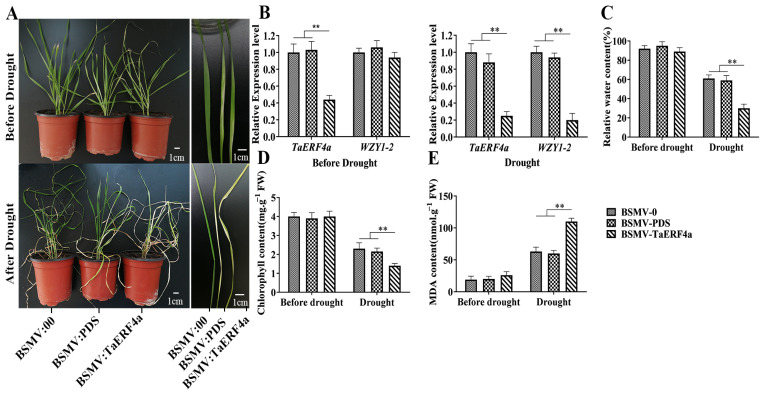
Performance of VIGS-mediated *TaERF4a*-silencing wheat under drought treatment; the phenotypes (**A**), *TaERF4a* and *WZY1-2* gene expression levels (**B**), relative water content (**C**), chlorophyll content (**D**), and malondialdehyde content (**E**) of wheat leaves treated with BSMV:00 (empty vector), BSMV:PDS, and BSMV:TaERF4a before and after drought treatment. Data are presented as means ± SD of three replicates (** *p* < 0.01).

BSMV-mediated virus-induced gene silencing (VIGS) method was also employed in Chinese spring wheat to investigate the function of the *TaERF4a* gene. After virus inoculation, wheat leaf photobleaching was observed in both the BSMV:PDS and BSMV:TaERF4a lines, indicating that the endogenous PDS gene was silenced. After 10 days of natural drought, the leaf wilting degree of the BSMV:TaERF4a line was more severe than that of the BSMV:0 and BSMV:PDS lines (Figure 3A). qRT-PCR analysis showed that the gene expression levels of *TaERF4a* and *WZY1-2* in the BSMV:TaERF4a lines were significantly lower than those in the other two lines (Figure 3B). Compared with the BSMV:0 and BSMV:PDS lines, the MDA content in wheat leaves of the BSMV:TaERF4a lines was significantly increased under drought stress, whereas the relative water content and chlorophyll content were significantly reduced (Figure 3C–E).

### 2.4. Transactivation Analysis of TaERF4a Protein

**Figure 4:**
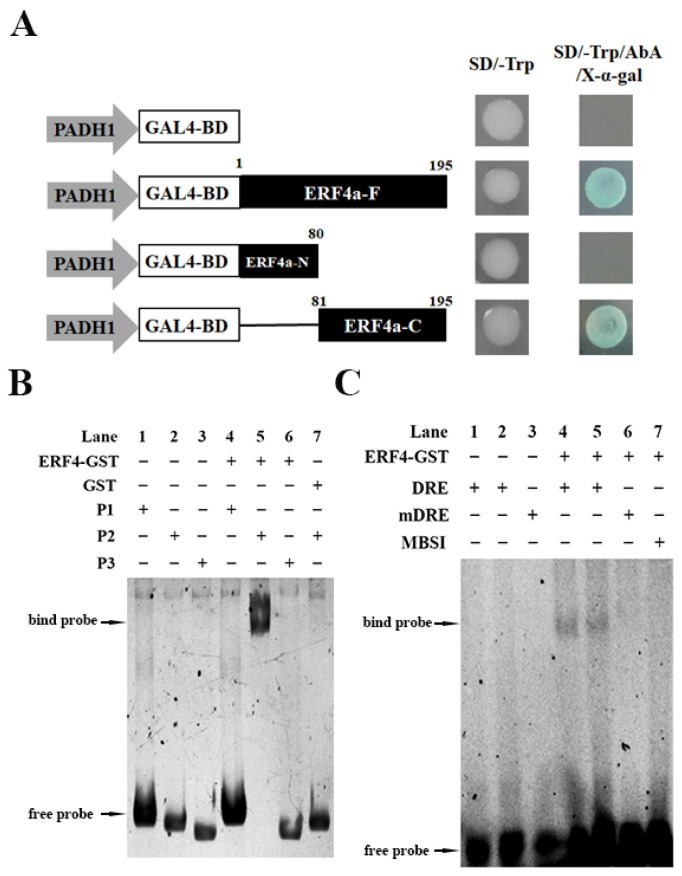
Transactivation activity assay of TaERF4a in yeast cells and its interaction with the promoter of the *WZY1-2* gene in vitro; (**A**) diagram of three fragments encoding the full-length, N-terminus (1–80 amino acids), and C-terminus (81–195 amino acids) of TaERF4a, which were, respectively, introduced into the pGBKT7 vector. Transactivation assays of fusion proteins were performed in the Y2H Gold yeast strain; (**B**) electrophoretic mobility shift assay of transcription factor TaERF4a binding with the P1 (−718 to −462 bp), P2 (−472 to −218 bp), and P3 (−220 to +15 bp) fragments of the *WZY1-2* promoter. TaERF4a-GST represents the purified fusion protein. GST was used as the negative control; and (**C**) EMSA analysis of TaERF4a binding to the DRE *cis*-acting element in the promoter segment of the *WZY1-2* gene. The *cis*-acting elements involved include the dehydration-responsive element (DRE: GCCGAC), myb-binding site I (MBSI: TAACTG), and mutant DRE (mDRE: GTCGCA).

To verify the interaction between TaERF4a and the dehydrin *WZY1-2* gene promoter, the bait yeast strains (Y1H Gold containing pAbAi-Pwzy1-2) transformed with the individual prey vector pGADT7-TaERF4a were able to grow on both SD/-Leu and SD/-Leu/AbA^200^ selective medium, demonstrating the interaction between TaERF4a and the *WZY1-2* gene promoter [20]. Moreover, the transactivation ability of TaERF4a was analyzed using the yeast assay system (Figure 4A). Three fragments encoding the full-length TaERF4a, TaERF4a-N (1–80 aa with AP2/ERF domain), and TaERF4a-C (81–195 aa) terminal were inserted into the pGBKT7 vector, respectively. These DNA-binding domain (BD) fusion plasmids were transformed into Y2H Gold yeast cells to confirm their ability to activate the transcription of the α-galactosidase reporter gene. The yeast transformants harboring pGBKT7-TaERF4a-F or pGBKT7-TaERF4a-C grew well and displayed blue colonies on SD/-Trp medium (synthetic dropout nutrient medium) supplemented with 20 μg/mL X-α-Gal and 200 ng/mL Aureobasidin A. In contrast, yeast cells containing pGBKT7-TaERF4a-N failed to grow on SD/-Trp/X-α-Gal/AbA^200^ selective medium.

### 2.5. EMSA Analysis of TaERF4a Binding to the WZY1-2 Promoter

The ERF gene shares at least one AP2/ERF domain, which is composed of 60 conserved amino acid residues and is involved in DNA binding with the *cis*-acting elements in the promoter of stress-responsive genes, including the GCC box, dehydration-responsive elements (DREs)/C-repeat elements (CRTs) [21,22]. Therefore, the binding site of TaERF4a transcription factor to the *WZY1-2* promoter was next determined. We performed the electrophoretic mobility shift assay (EMSA) to investigate the interaction and binding motif of TaERF4a to the *WZY1-2* promoter in vitro. The *WZY1-2* promoter was divided into three segments, P1, P2, and P3. As shown in Figure 4B, the transcription factor TaERF4a incubated with the P2 fragment showed an obvious gel migration band, indicating that the TaERF4a protein could strongly bind to the P2 fragment in vitro, whereas no significant affinity was observed after incubation with the P1 or P3 fragment. In addition, we found the DRE motif present in the P2 promoter region, which is a major target of AP2/ERF transcription factors in plant abiotic stress responses [23,24]. Next, we analyzed the binding ability of TaERF4a to the DRE *cis*-element. As shown in Figure 4C, TaERF4a did not bind to the mutated DRE probe or other motifs, confirming its specific binding to the DRE *cis*-acting element.

### 2.6. Dual-Luciferase Assay of TaERF4a in Plants

**Figure 5:**
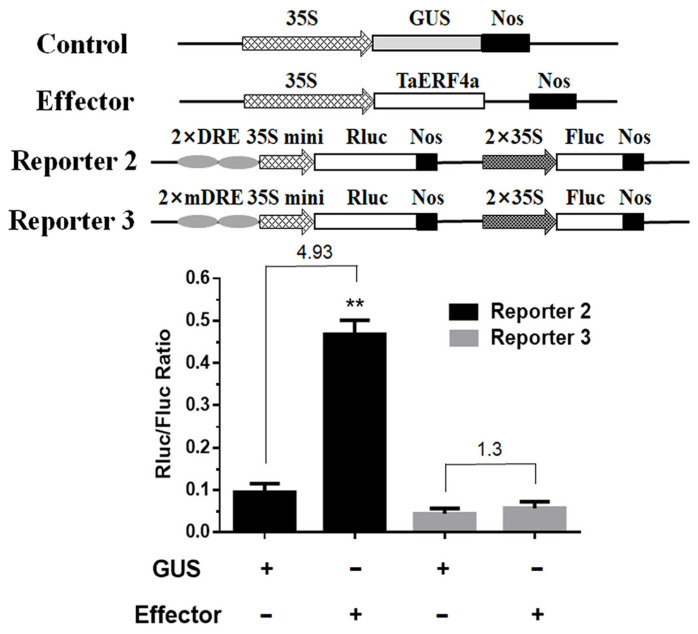
Transient luciferase activity assay of the DRE motif bound by TaERF4a in *N. benthamiana* leaves. Schematic diagrams of effector and reporter constructs used in the assay. The described constructs were transformed into *Agrobacterium tumefaciens* strain GV3101 and then infiltrated into *N. benthamiana* leaves. The experiments were performed with at least three biological repeats. Data are presented as means ± SD of three replicates (** *p* < 0.01).

We demonstrated that TaERF4a can positively regulate the expression of the *WZY1-2* dehydrin gene by using the dual-luciferase transient expression assay [20]. To test whether TaERF4a depends on the DRE *cis*-element to activate the *WZY1-2* gene expression, a luciferase assay was also performed *in planta*. After mixing *A. tumefaciens* harboring the p35S::TaERF4a (effector vector) with those containing the 2 × DRE-minimal 35S promoter::Rluc (reporter vector 2) or the mutant DRE *cis*-element 2 × DREm-minimal 35S promoter::Rluc (reporter vector 3), the mixture was instantaneously transformed into *N. benthamiana* leaves by agro-infiltration and then subjected to luciferase activity analysis (Figure 5). We found that, in the plants transformed with reporter vector 2, the expression of TaERF4a resulted in a more than 4.9-fold increase in luciferase activity (Rluc/Fluc ratio) compared with the GUS control. In contrast, in the plants transformed with reporter vector 3 containing the mutant DRE element, luciferase activity showed a similar level to that of the control.

## 3. Discussion

Plants evolved a variety of defense mechanisms to counter adverse environmental factors, in which dehydrins play an important role in the plant stress response. We previously isolated and characterized the full-length cDNA, genomic, and promoter sequences of the SK_3_-type dehydrin WZY1-2 in the Zheng yin 1 cultivar of *Triticum aestivum* [23]. Moreover, TaERF4a was identified as a potential upstream regulator of the *WZY1-2* gene through yeast one-hybrid assay. TaERF4a responds to abiotic stress and positively regulates the expression of WZY1-2 dehydrin.

Transcription factors have multiple functions not only in plant growth and development, but also in abiotic stress responses. Studies have shown that the AP2/ERF family of transcription factors is widely involved in regulating plant responses to various abiotic stresses. Kumar et al. found that rice plants overexpressing *Os-AP2/ERF-N22* showed higher relative water content, membrane stability index, osmotic potential, stomatal conductance, wax content, and radical scavenging activity [25]. Huang et al. reported that *OsERF19*-overexpressing lines increased the tolerance of rice plants to salt stress and upregulated the expression of stress-responsive genes, including *OsLEA3*, *OsNHX1*, *OsHKT6*, and *OsOTS1* [26]. Under heat stress, AP2/ERF transcription factor ERF1 positively regulates heat tolerance in plants by binding to DRE *cis*-elements in heat-responsive genes, such as *HSFA3* and *HSPs*, and activating their expression [12]. In addition, studies have shown that the *TaERF1* gene can participate in JA, ET, and ABA signal transduction pathways and activate the expression of stress resistance genes. Furthermore, *TaERF1*-overexpressing *Arabidopsis* significantly increased its resistance to drought, high salt, and high temperature, while reducing leaf water loss by decreasing stomatal aperture [27].

**Figure 6:**
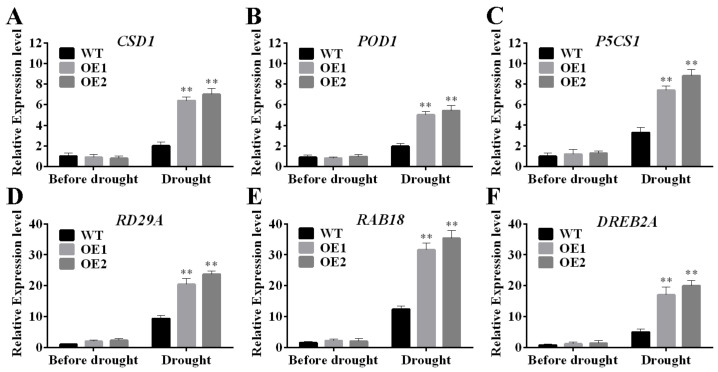
The expression levels of stress-responsive genes *CSD1* (**A**), *POD1* (**B**), *P5CS1* (**C**), *RD29A* (**D**), *RAB18* (**E**) and *DREB2A* (**F**) in WT and transgenic *Arabidopsis* plants under normal and drought treatments. Data are presented as means ± SD of three replicates (** *p* < 0.01).

In this study, we investigated the role of TaERF4a transcription factor in response to plant abiotic stress. Proline, MDA, SOD, and chlorophyll content were measured in WT and transgenic lines under drought stress. These physiological indices in transgenic plants were superior to those in WT plants. In contrast, VIGS-mediated *TaERF4a*-silencing plants exhibited a drought-sensitive phenotype and lower biochemical indices after exposure to drought stress. These results indicate that TaERF4a has a positive effect on plant drought tolerance. As is well known, drought is one of the most common abiotic stresses for plants, negatively affecting water and nutrient uptake and impeding root growth and development. Plant roots grown in soil can perceive different environmental stresses and adapt their architecture accordingly [28]. Notably, *TaERF4a*-overexpressing *Arabidopsis* produced longer roots than WT plants under drought stress conditions. Furthermore, studies reported that the AP2/ERF transcription factors improve stress resistance by regulating the osmotic potential and reactive oxygen species scavenging capacity. For example, BpERF13 upregulates superoxide dismutase (SOD) and peroxidase (POD) genes, thereby enhancing the tolerance of transgenic plants to cold stress [29]. Meanwhile, AP2/ERF transcription factors (TFs) are involved in stress responses by regulating the expression of stress-related genes. For instance, AgDREB1 and AgDREB2 act as transcriptional activators to regulate downstream genes by binding to the corresponding DRE/CRT element, thus enhancing the stress resistance of celery [30]. To elucidate the putative mechanism of TaERF4a on drought stress tolerance, qRT-PCR analyses were performed to examine the expression patterns of stress-responsive genes involved in the ROS-scavenging system, proline biosynthesis, and other pathways in both WT and *TaERF4a*-overexpressing plants before and after drought stress treatment (Figure 6). The expression levels of drought stress-related and ROS scavenging genes, including *copper/zinc superoxide dismutase 1* (*CSD1*), *peroxidase* (*POD1*), *Δ-1-pyrroline-5-carboxylate synthase1* (*P5CS1*), *responsive to dehydration 29A (RD29A)*, and *responsive to ABA 18* (*RAB18*), were upregulated to improve ROS scavenging ability and decrease stress-induced damage. Moreover, the expression levels of these genes were markedly higher in transgenic plants than in WT plants. The expression of these stress resistance genes is consistent with the stress tolerance phenotype of transgenic plants under drought conditions. Therefore, these results suggest that TaERF4a may function positively to activate the expression of stress signaling-related genes in response to drought stress.

Transcription factors participate in plant stress responses by regulating gene expression through binding to downstream stress response gene promoters [31]. Yeast one-hybrid and EMSA assays demonstrated that the TaERF4a protein binds and interacts with the *WZY1-2* gene promoter. A luciferase assay showed that TaERF4a positively regulates the expression of the dehydrin *WZY1-2* gene, as reported in our previous study. In VIGS-mediated transient expression analysis, the *WZY1-2* gene expression level was significantly reduced in BSMV:TaERF4a lines under drought stress compared to the control (Figure 3B). Furthermore, TaERF4a transcription factor binds the DRE *cis*-acting element in the *WZY1-2* promoter to regulate its expression. Thus, these results indicate that TaERF4a is a potential positive regulator of WZY1-2 expression.

As dehydrins are localized to the nucleus, they can also protect nucleic acids and proteins by binding to them against damage from ROS under stress conditions [32]. In our last report, GFP fusion and BiFC assays demonstrated that the dehydrin WZY1-2 localizes to the nucleus as a homodimer [33]. The ability of dehydrin to form dimers at specific subcellular locations *in planta* can help strengthen its biological function [34]. Additionally, our earlier research revealed that the expression of *WZY1-2* dehydrin in wheat increases with drought severity at the seedling stage, suggesting its role in drought stress response [35].

**Figure 7:**
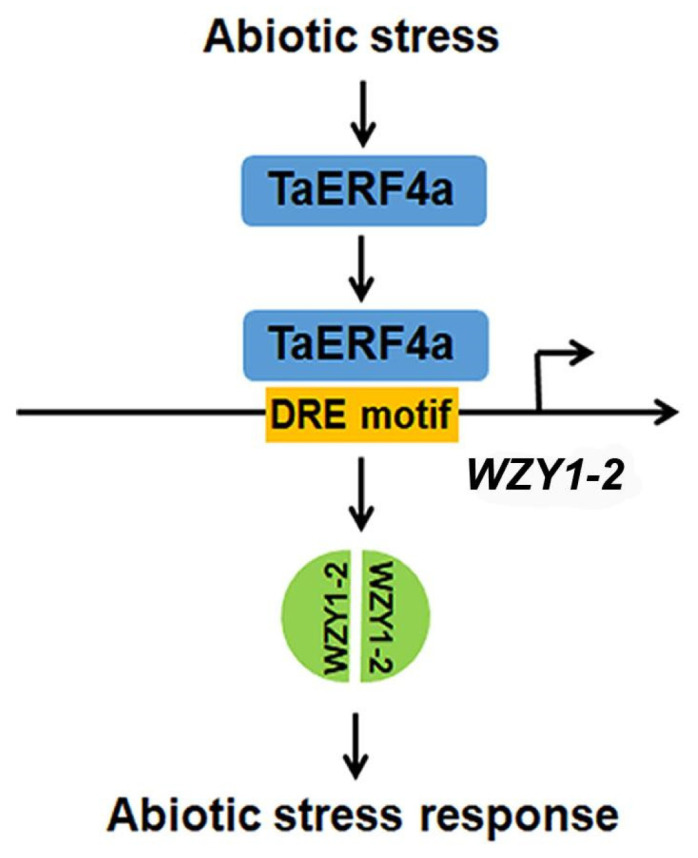
A working model of TaERF4a and WZY1-2 in abiotic stress response.

Taken together, we propose a potential model to explain the regulatory mechanism underlying the stress resistance of the WZY1-2 dehydrin in wheat. The transcript level of *TaERF4a* gene is upregulated under abiotic stress. TaERF4a activates *WZY1-2* gene transcription by directly binding to the DRE *cis*-acting element in the *WZY1-2* promoter. The nucleus-localized WZY1-2 then forms a homodimer to mediate the response to abiotic stress in wheat (Figure 7).

## 4. Materials and Methods

### 4.1. Plant Growth Conditions

Wheat (*Triticum aestivum* cv. Chinese spring), *Arabidopsis thaliana* (ecotype Col-0), and tobacco (*Nicotiana benthamiana*) were grown in a growth chamber at 22 °C and 60% humidity under 16 h/8 h light/dark cycles with a constant white light intensity of 250 μmol photons·m^−2^·s^−1^.

### 4.2. Acquisition of TaERF4a-Overexpressing Transgenic Arabidopsis thaliana

The amplified TaERF4a coding sequence was cloned into the *Kpn* I/*Hind* III digested plant expression vector pCAMBIA1302. The recombinant plasmid pCAMBIA1302:TaERF4a was then transformed into *Agrobacterium tumefaciens* strain GV3101 using the electric shock method. *Agrobacterium*-mediated transformation of *Arabidopsis* was performed via the floral dipping method to generate TaERF4a overexpression in transgenic plants. Homozygous T3 progeny of the transgenic *Arabidopsis* plants was selected for further analysis.

### 4.3. VIGS-Mediated TaERF4a Gene Silencing in Wheat

In vitro transcripts of the Barley stripe mosaic virus vectors BSMV-α and BSMV-β were mixed with the transcripts of BSMV-γ (negative control), BSMV-γ-PDS (positive control), or BSMV-γ-PDS-TaERF4a (experimental group) at an equal ratio of 3 μL each. The transcript mixture was then mixed with 91 μL FES buffer and used for viral inoculation on tobacco leaves. The transcription method followed the protocol of the T7 High Yield RNA Transcription kit (Novoprotein, Suzhou, China), and plant inoculations were performed as previously described [15]. The experiment was repeated three times, and the results were averaged.

### 4.4. Quantitative Real-Time PCR

Trizol was used to isolate total RNA from the sample, and first-strand cDNA was synthesized from total RNA (1 μg) by using TransScript One-step cDNA Synthesis SuperMix (TransGen, Beijing, China) according to the manufacturer’s instructions. The reaction system was as follows: 2 × TransStart Tip Green qPCR SuperMix 10 μL, Forward Primer (10 μmol/L) 0.4 μL, Reverse Primer (10 μmol/L) 0.4 μL, cDNA template (20 ng/μL) 1 μL, ddH_2_O 8.2 μL. The qPCR reaction was pre-denatured at 94 °C for 4 min, 40 cycles of 94 °C for 30 s, and 60 °C for 30 s, followed by a melting curve analysis. All reactions were performed in triplicate to ensure repeatability of the results, and data analysis was conducted using the 2^−ΔΔCt^ method. Primer sequences are listed in Table S1.

### 4.5. Performance of TaERF4a-Overexpressing Arabidopsis thaliana Plants Under Drought Conditions

Seeds of the wild-type (WT) and T3 homozygous *TaERF4a*-overexpressing lines were sown on 1/2 MS agar medium and incubated at 22 °C under 16 h light/8 h dark cycles for 3 days. The germinated seedlings were then transferred to the same medium supplemented with 10% PEG 6000 and grown for another week. Additionally, to investigate the drought tolerance of the transgenic lines, both WT and transgenic seedlings grown under normal conditions for 3 weeks were subjected to water deficit by withholding water for 12 days. The rehydration assay was carried out over the following three days.

### 4.6. Measurements of Malondialdehyde (MDA), Water Loss Rate, and Chlorophyll Content Physiological Indices

MDA content, SOD activity, and proline content were quantified using commercial detection kits (Jiancheng Bioengineering Institute, Nanjing, China). The water loss rate, relative water content, and chlorophyll content of wheat leaves were measured as previously described [36,37].

### 4.7. Transactivation Analysis of TaERF4a

The transactivation ability of the TaERF4a protein was analyzed using the Y2H Gold yeast strain. The full-length and two truncated fragments of TaERF4a fused with the GAL4 DNA-binding domain of the pGBKT7 plasmid were transformed into Y2H Gold cells, respectively, and then grown on SD-Trp/X-α-Gal/AbA medium to confirm their transactivation ability.

### 4.8. Electrophoretic Mobility Shift Assay

The binding of transcription factor TaERF4a with the fragment of WZY1-2 promoter was analyzed by electrophoretic mobility shift assay (EMSA). The WZY1-2 promoter sequence was divided into three segments: P1 (−718 to −462 bp), P2 (−472 to −218 bp), and P3 (−220 to +15 bp). The EMSA was performed using the LightShift^®^ Chemiluminescent EMSA Kit (Thermo Fisher Scientific, Waltham, MA, USA) according to the manufacturer’s protocol. The WZY1-2 promoter fragment was incubated with purified TaERF4a protein at room temperature for 30 min and then separated by 6% native polyacrylamide gel electrophoresis.

### 4.9. Dual-Luciferase Transient Expression Assay

To investigate whether TaERF4a relies on the DRE element to activate *WZY1-2* gene expression, a dual-luciferase assay was conducted in *N. benthamiana*. The effector vector pCAMBIA1301:TaERF4a was transformed into *Agrobacterium* tumefaciens strain GV3101. Reporter vector 2 was constructed by inserting two copies of the DRE *cis*-element upstream of the minimal 35S promoter and ligating it to the dual-luciferase reporter vector pC0390-RUC. Reporter vector 3 was constructed similarly but with two copies of a mutant DRE *cis*-element. An equal mixture of *Agrobacterium* transformants containing the effector vector and either reporter vector 2 or vector 3 was injected into tobacco leaves through agroinfiltration. The plants were cultured under normal light conditions for 2 days after being incubated in darkness for 8 h. The infiltrated leaves were collected and frozen in liquid nitrogen for subsequent luciferase activity (RLuc/Fluc) analysis using the Dual-Luciferase^®^ Reporter Assay System (Promega, Fitchburg, WI, USA).

## Data Availability

The raw data supporting the conclusions of this article will be made available by the authors on request.

## Supplementary Materials

The following supporting information can be downloaded at: https://www.mdpi.com/article/10.3390/plants14081214/s1, Table S1: Primer Sequences; Table S2: Sequences of TaERF4a and the WZY1-2 gene promoter.

## Abbreviations

DHNs: dehydrins; SOD, superoxide dismutase; MDA, Malondialdehyde; qRT-PCR, quantitative real-time polymerase chain reaction; ROS, reactive oxygen species; Pwzy1-2, promoter of the dehydrin WZY1-2 gene; EMSA, electrophoretic mobility shift assay; VIGS, virus-induced gene silencing; AbA, Aureobasidin A; DRE, dehydration-responsive binding element; Rluc, Renilla luciferase; Fluc, Firefly luciferase.
