# Supplementary material for: Exploring the Role of TaERF4a in Enhancing Drought Tolerance and Regulating Dehydrin WZY1-2 Gene Expression in Wheat
Source: Plants (Basel). 2025 Apr 15;14(8):1214. doi: 10.3390/plants14081214 (PMC12030695; doi:10.3390/plants14081214)
Supplement: Supplementary file 1 [file plants-14-01214-s001.zip › plants-3543125-supplementary.pdf]

Supplemental Table S1&S2

Table S1 List of Primers used in this study

Primer name	Primer sequence (5′-3′)
Pwzy1-2 F	acgaaatccaccacgaactc
Pwzy1-2 R	gatcgagatcgatcggtgca
Pwzy1-2 F1	cgaagcttacgaaatccaccacgaactc
Pwzy1-2 R1	atgagctcgatcgagatcgatcggtgca
TaERF4a F	atgcgcaaggcgagg
TaERF4a R	gaggcggagcagcgt
TaERF4a F1	actcttgaccatggtagatctatgcgcaaggcgagg
TaERF4a R1	ctcgcccttgctgtcagatctgaggcggagcagcgt
TaERF4a F2	ttccaggggcccctgggatccatgcgcaaggcgagg
TaERF4a R2	ctcgagtcgacccgggaattcgaggcggagcagcgt
Pwzy1-2 F2	aacgaaatccaccac
Pwzy1-2 R2	ctctctccgtggcgt
Pwzy1-2 F3	cacggagagaggggg
Pwzy1-2 R3	acaagcaggaaagaa
Pwzy1-2 F4	tctttcctgcttgtg
Pwzy1-2 R4	gatcgagatcgatcg
DRE F	ttatttccggccgacacgct
DRE R	agcgtgtcggccggaaataa
mDRE F	ttatttccggtcgacacgct
mDRE R	agcgtgtcgaccggaaataa
MBSI F	gagcgtaactgcccaccact
MBSI R	agtggtgggcagttacgctc
Zl Pwzy1-2 F	gcaggtcgacggatccccgggacgaaatccaccacg
Zl Pwzy1-2 R	ggtggactcctcttagaattcgatcgagatcgatcg
qActin F	ctccctcacaacaaccgc
qActin R	taccaggaacttccatac
qWZY1-2 F	ggaagagcccgaggtcaag
qWZY1-2 R	gcttttccttgagcccctt
qTaERF4a F	gacgagaatagcaaggattcgt
qTaERF4a R	gcaaaagagaacaccacaagat

qAtPOD1 F	tctgaccgttcaagaaatgg
qAtPOD1 R	tggagcaacccgtaaccgtg
qAtRD29A F	acgtttgctccaagtggtga
qAtRD29A R	cctccaacgttatcggggtc
qAtP5CS1 F	ttgtgatcccaagaggaagc
qAtP5CS1 R	cgctttgccatatccgtatc
qAtCSD1 F	actgccaccttcacaatcactg
qAtCSD1 R	gctttagccctggagaccaatg
qAtRAB18 F	cagcagcagtatgacgagta
qAtRAB18 R	cagttccaaagccttcagtc
qAtDREB2A F	caggctttggcttggtactttc
qAtDREB2A R	cacaaccaggagtctcaacagt

Table S2 Sequences of *TaERF4a* and the *WZY1-2* gene promoter

Gene	Sequence
TaERF4a	ATGCGCAAGGCGAGGCCGCCGCAGCCCCAGCCGCAGCCGTCGCCGGAGATCCGGTACCG<br>CGGCGTGCGGAAGCGCCCCTCGGGCCGCTACGCCGCCGAGATCCGGGACCCGGCCAAGA<br>AGACGCCGATCTGGCTGGGCACCTTCGACTGCGCCGAGGACGCCGCCCGCGCCTACGAC<br>TCCGCCGCCCGATCCCTCCGCGGGCCCACCGCCCGCACCAACTTCCCGCCCTCCTCCGCC<br>ACGCAGCCGCCGCCGCGCCCTCCCCCTCCCCCCGCGGCGGCCGCCGCGACCGCCACGAC<br>CAGCCAGAGCAGCACCGTCGAGTCCTGGAGCGGCGGCGGGCCCCGCGCCCCCGCCAGGG<br>CCCGCAGCGCCGCCCGAGCGGGCACGGCCGAGGAAGGGGAGGAGGACTGCCGCAGCTA<br>CTGCGGCTCCTCCTCCTCCGTCCTCCTCTGCGAGGATGGGGACGACGCGGCCGCCTCCCG<br>CACCCCGCTGCCCTTCGATCTGAACATGCCGCCCCCACAGGACGGGGCCCTTGACGCCGC<br>GGCCGCCGAGGCTGATCAGATGACCTGCCGCTACGACACGCTGCTCCGCCTCTAG
WZY1-2 Promoter	AACGAAATCCACCACGAACTCTTATCTTCGTCCGCTACCGGTGATTGAGCATCCGGAAGA<br>ATCAATGTTAATGCCACACAACCACTTAAATAAACCCCGGCCAATCGTGTGTGGACAAA<br>ATGGCTCACGCCACTGTTCCAAAATCGATCAATCACTTAATCAATTGGTTGAATTATCTC<br>CTAAAGTATTGCCGGAGAGTGACGGGCGTGGACAAGCGTCCCGTCGGCACCGGATGGGA<br>GGAAACGCCACGGAGAGAGGGGGGAGGGAGCACCGCACCGACCATTCGCACCGGGCCA<br>CGCCTCGGTGAGCGTAACTGCCCACCACTCCACATGGCGCCCGCCGCCCTCTCCCGATGC<br>CGTCGATGGCGCGTCCTCCACGTCGGACTGACCGCCCCACGACATGCGTCGGGGCTCCA<br>CTGGCTCACGCGCTGCCCCGTCACGCCATTATTTCCGGCCGACACGCTGTCACTCGCGAC<br>GTTGAACCGTCCTTCTTTCCTGCTTGTGTCCTCACGTACAGCGCTACAGAGCACAGATGT<br>ACACCTTTCGGCAGCGGCTCCCGATCGAACTCCTTGACAGCGGCTATATAAGGAAGCCTC<br>TTGGCCCAGACACCTTCATCAGTCACAAAGCCAAAGCAAAAGCCACAAGCCAAGAACCA<br>ATACTTGATCTGTTGTTTCCTTTAGCTCCCGGAAGACTTTTAGCTGCACCGATCGATCTCG<br>ATC
